# *In vivo* antitumor effect of endostatin-loaded chitosan nanoparticles combined with paclitaxel on Lewis lung carcinoma

**DOI:** 10.1080/10717544.2017.1378938

**Published:** 2017-09-21

**Authors:** Fang Xie, Rui-Lin Ding, Wen-Feng He, Zong-Jun-Lin Liu, Shao-Zhi Fu, Jing-Bo Wu, Ling-Lin Yang, Sheng Lin, Qing-Lian Wen

**Affiliations:** a Department of Oncology, Affiliated Hospital of Southwest Medical University, Luzhou, Sichuan, China;; b Experiment and Training Center, Sichuan College of Traditional Chinese Medicine, Mianyang, Sichuan, China

**Keywords:** Endostatin, nanoparticles, chitosan, paclitaxel, lung cancer

## Abstract

The purpose of this study was to prepare endostatin-loaded chitosan nanoparticles (ES-NPs) and evaluate their antitumor effect when combined with paclitaxel (PTX) on Lewis lung carcinoma (LLC) mouse xenografts. ES-NPs were prepared by ionic cross-linking. Characterization of the ES-NPs included size distribution, drug-loading efficiency (DL), and encapsulation efficiency (EE). An *in vitro* release test was also used to determine the release behavior of the ES-NPs. A subcutaneous LC xenograft model of C57BL/6J mice was established. The mice were randomly divided into six groups: control (0.9% NaCl), ES, PTX, ES-NPs, ES + PTX, and ES-NPs + PTX. The tumor volume was dynamically measured for the duration of the experiment. Immunohistochemistry was performed to determine the Ki-67 and microvascular density (MVD) in each group. Serum vascular endothelial growth factor (VEGF) and ES levels were determined by enzyme-linked immunosorbent assay (ELISA). ES-NPs were successfully synthesized and had suitable size distribution and high EE. The NPs were homogenously spherical and exhibited an ideal release profile *in vitro. In vivo*, tumor growth was significantly inhibited in the ES-NPs + PTX group. The tumor inhibitory rate was significantly higher in the ES-NPs + PTX group than in the other groups (*p* < .05). The results of the immunohistochemical assay and ELISA confirmed that ES-NPs combined with PTX had a strong antiangiogenic effect. ES-NPs can overcome the shortcomings of free ES, such as short retention time in circulation, which enhances the antitumor effect of ES. The antitumor effect was more pronounced when treatment included PTX and ES-loaded NPs.

## Introduction

Lung cancer is the leading cause of cancer death in developed and developing countries, and has surpassed breast cancer as the leading cause of cancer death in developed countries (Li et al., 2013; Torre et al., 2015; Siegel et al., 2017). Chemotherapy for non-small cell lung carcinoma (NSCLC) consists of platinum-based drugs combined with cytotoxic drugs like paclitaxel (PTX) (Azzoli et al., 2012). However, inadequate tumor vascularization leads to the inefficient distribution of drugs in tumor cells, which is an important obstacle to anticancer therapies (Shi et al., 2013).

The vascular normalization theory posits that using inhibitors of vascular endothelial growth factor (VEGF) signaling in combination with chemotherapeutics can increase antitumor efficacy (Ferrara & Kerbel, 2005; Takahashi et al., 2012; Klose et al., 2016). Clinical trials have shown that the chemotherapeutic agent PTX combined with the antiangiogenic peptide endostatin (ES) improves clinical therapeutic efficacy in advanced NSCLC patients without unacceptable toxicity (Han et al., 2011; Sun et al., 2016). ES is a 20 kDa internal fragment of the carboxy-terminus of collagen XVIII. It was identified as an antiangiogenic molecule in 1997 (O'Reilly et al., 1997). ES targets VEGF and acts against solid tumors by inhibiting endothelial cell proliferation, migration, and vessel formation (Kim et al., 2002; Ling et al., 2007; Chen & Hu, 2011). Nevertheless, ES has several disadvantages that include short half-life and instability, which limit its clinical application (Torchilin & Lukyanov, 2003).

Nanoparticles (NPs) offer an alternative strategy to overcome these drawbacks and increase the antitumor effect (Singh & Lillard, 2009). Chitosan, the *N*-deacetylation product of chitin, is characterized by low toxicity, good biodegradability, and superb biocompatibility, whose derivable reactive groups can be functionalized with proteins and peptides. Additionally, the production of chitosan-based NPs is relatively simple and mild, and avoids the use of organic solvents and high temperatures (Lee et al., 1995; Al-Qadi et al., 2012; Rampino et al., 2013; Swierczewska et al., 2016). Chitosan is a polycationic biopolymer comprised of amine and hydroxyl groups. The polymer can form a gel forming upon contact with specific multivalent polyanions, such as sodium tripolyphosphate (TPP). Inter- and intramolecular linkages between the phosphate groups of TPP and the amino groups of chitosan form NPs. The main advantage of CS-NPs is that they protect their payload from premature chemical or enzymatic degradation (Calvo et al., 1997; Nasti et al., 2009). Furthermore, because of their high surface to volume ratio, CS-NPs can transport a large payload with fewer materials (Swierczewska et al., 2016).

This study describes the preparation and characterization of ES-loaded chitosan-TPP NPs using the ionotropic gelation method. Moreover, we explored the *in vivo* antitumor effect of these NPs combined with PTX.

## Materials and methods

### Materials

ES (11.2 mg/mL, Mw = 20 kDa) was provided by Shandong Simcere Medgeen Bio-Pharmaceutical Co. Ltd. (China). Low molecular weight chitosan (Mw = 70 kDa, Mw/Mn = 1.5, deacetylation degree 92%), TPP, phosphate-buffered saline (PBS), and trehalose were purchased from Sigma-Aldrich Inc. (St. Louis, MO). PTX (5 mL; 30 mg injectable solution) manufactured by the Taiji Group Co., Ltd. (China) was provided by Southwest Medical University (China). Polyclonal ntibodies to Ki67 and CD31 were purchased from Bioworld Technology Co. Ltd. (China). All other chemicals, including sodium hydroxide, glacial acetic acid, and paraformaldehyde, were of analytical grade and were used as received.

### Cell culture

Lewis lung carcinoma (LLC) cells were provided by the Experimental Medicine Center, Affiliated Hospital of Southwest Medical University (China). LC cells were grown in Dulbecco's modified Eagle’s medium (DMEM; HyClone, Thermo Scientific, Waltham, MA) supplemented with 10% fetal calf serum (HyClone, Thermo Scientific), 100 IU/mL of penicillin G sodium, and 100 mg/mL of streptomycin sulfate. The cells were maintained at 37 °C in an incubator with 95% air and 5% CO_2_ in a humidified atmosphere.

### Animals

Female C57BL/6J mice (4–5 weeks of age) were purchased from the Laboratory Animal Center of the Chongqing Municipality (China). Mice were kept in a specific-pathogen-free (SPF) laminar air flowbox and were fed with sterile food pellets and water *ad libitum*. All animal care and experimental procedures were approved and performed according to the Institutional Animal Care and Use Guidelines. Animal experiments were approved by the Institutional Animal Care and Treatment Committee of Southwest Medical University (China).

### Preparation of ES-NPs

NPs were synthesized by a simple ionic cross-linking method as described previously (Rampino et al., 2013). Chitosan 40 mg dissolved in 1% (v/v) acetic acid (1 mg/mL, pH 5) and TPP (1 mg/mL) was prepared in deionized water. TPP solution (8 mL) was added dropwise to the chitosan solution mixed in 500 mL of ES with magnetic stirring at room temperature (RT) for 2 h to form NPs, as described previously (Ding et al., 2017). The NPs were collected by centrifugation in an Allegra 64R centrifuge (Beckman-Coulter, Brea, CA) at 13,000 rpm for 30 min at 4 °C. The precipitate was resuspended in deionized water and lyophilized by freeze-drying at 55 °C for 48 h using trehalose as the cryoprotectant. Finally, the dry ES-loaded NPs were weighed using an electronic weighing scale sterilized by 25 kGy of ^60^Co gamma irradiation.

### Characterization of ES-NPs

Size distribution and zeta-potential of ES-loaded NPs in distilled water were measured by dynamic light scattering (DLS) using a NanoBrook 90Plus Zeta apparatus (Brookhaven Instruments Corp., Holtsville, NY). The surface morphology of the NPs was investigated by transmission electron microscopy (TEM) using a model G2 F20 microscope (Tecnai, Hillsboro, OR).

### Drug-loading (DL) and encapsulation efficiency (EE) of ES-NPs

Supernatants obtained from the purification of the ES-NPs were collected to determine particle loading and EE. The concentration of ES in the supernatant was measured using a Pierce Bicinchoninic acid Protein (BCA) assay (Beyotime Biotechnology, China) (Mukherjee et al., 2008; Xu et al., 2014). The results of the assay were validated using purified ES with a detection limit of 0.5–280 mg/mL. The amount of ES was calculated according to the standard curve (*R*^2^ = 0.9992). The DL and EE of the ES-NPs were expressed as follows:

$$DL\%\;=\frac{Total\;amount\;of\;ES\;added-Free\;amount\;of\;ES}{ES-loaded\;NPs\;dry\;weight}\times 100\%$$

$$EE\%=\frac{Total\;amount\;of\;ES\;added-Free\;amount\;of\;ES}{Total\;amount\;of\;ES\;added\;}\times 100\%$$

### *In vitro* drug release and stability study of the ES-NPs

*In vitro* release of ES from the ES-NPs was analyzed in PBS having a pH of 7.4 and 5. ES-NPs were precisely weighed in 2 mL of PBS, placed in test tubes, and magnetically stirred at 37 °C. At defined times, samples were centrifuged at 13,000 rpm for 30 min at 4 °C, the supernatants were collected and each pellet was resuspended in 2 mL of fresh medium. The amount of ES released from the CS-NPs was evaluated using the BCA assay and the curve of ES release from the ES-NPs was plotted. In addition, to detect the stability of the carriers, the change in NP diameter in serum or PBS was examined over 72 h. The formulation stability of ES-NPs in PBS with or without 10% mouse serum, incubated at 4 °C, RT, or 37 °C was evaluated at pre-determined times by DLS as described above after ultrasonic processing. Formulations were deemed stable if no changes in particle size and no visual destabilization, such as creaming, phase separation, or presence of compact aggregates, were observed.

### *In vivo* anti-tumor efficacy of PTX combined with ES-NPs

The subcutaneous lung cancer model was established by injecting 100 mL suspension of LLC cells (1 × 10^7^ cells/mL) into the right armpit of C57BL/6J mice. The cells were allowed to grow for 2 weeks until the tumors were approximately 200 mm^3^ in volume. The tumor-bearing mice were randomly assigned to six groups (*n* = 10 each): control, ES, PTX, ES NPs, ES + PTX, and ES-NPs + PTX. According to clinical doses used in humans and the results from a previous study (Yu et al., 2015), 0.9% NaCl (0.2 mL/day), ES (10 mg/kg), and ES-NPs containing the same amount of ES were administrated intraperitoneally (i.p.) once daily for 14 consecutive days, with PTX (10 mg/kg) injected on the first day. Mice were sacrificed by cervical dislocation on day 21 and the tumor tissues and blood samples were collected for further analysis. During the treatment, tumor size (length and width) was measured using calipers every 2 days. Tumor volumes were calculated with the formula *V* = *a* × *b*^2^ × 0.5 (Tentler et al., 2010), where *V* is the tumor volume, *a* is the longest axis, and *b* is the perpendicular shorter tumor axis. A tumor growth curve was plotted based on tumor size and length of survival, in days, after treatment. The tumor volume inhibition rate on day 21 was calculated according to the following equation:

$$Inhibition\;rate\;\%=\left(1-\frac{(Volume\;Day\;1\;treatment\;group\;-\;Volume\;Day\;21\;treatment\;group)\;}{(Volume\;Day\;1\;control\;group-\;Volume\;Day\;21\;control\;group)}\right)\;\times \;100\%$$

### Enzyme-linked immunosorbent assay (ELISA) analysis

Blood samples were collected in Eppendorf tubes from each group. After immediate centrifugation (1800*g*) for 10 min at 4 °C, plasma was separated and immediately frozen at 80 °C until analysis. Plasma ES and VEGF levels were measured by an ELISA kit according to the manufacturer’s instructions (RayBiotech Inc., Norcross, GA). PBS was used as the control. Absorbance was measured at 450 nm. ES and VEGF concentrations were calibrated with ES and VEGF standard curves.

### Immunohistochemistry

Tumor tissue samples harvested from the sacrificed mice were fixed in 10% formalin, paraffin-embedded, and sectioned. Tissue sections 5 mm in thickness were dewaxed and incubated with 0.01 M sodium citrate for antigen retrieval. The slides were rinsed in PBS and incubated overnight at 4 °C with rabbit antimouse CD31 primary antibody (Bio-World, Dublin, OH). Biotinylated goat anti-rabbit anti-immunoglobulin G (IgG) was used as the secondary antibody. Steps were then performed using the immunostaining kit, following the manufacturer’s instructions. Quantification of microvessel density (MVD) expressed as the mean value of the area of microvessels in five CD31-highly positive hotspots at low magnifications (100×). Microvessels were counted in these areas at a magnification of 400×, and the average number of microvessels was recorded, as reported previously (Gao et al., 2009).

### Statistical analyzes

Statistical analyzes were carried out using SPSS 23.0 software (SPSS Inc., Chicago, IL), with Student’s *t*-test for two groups or one-way ANOVA for multiple groups. Means were considered significantly different when *p* < .05.

## Results

### Characteristics of ES-loaded NPs

**Figure 1.**
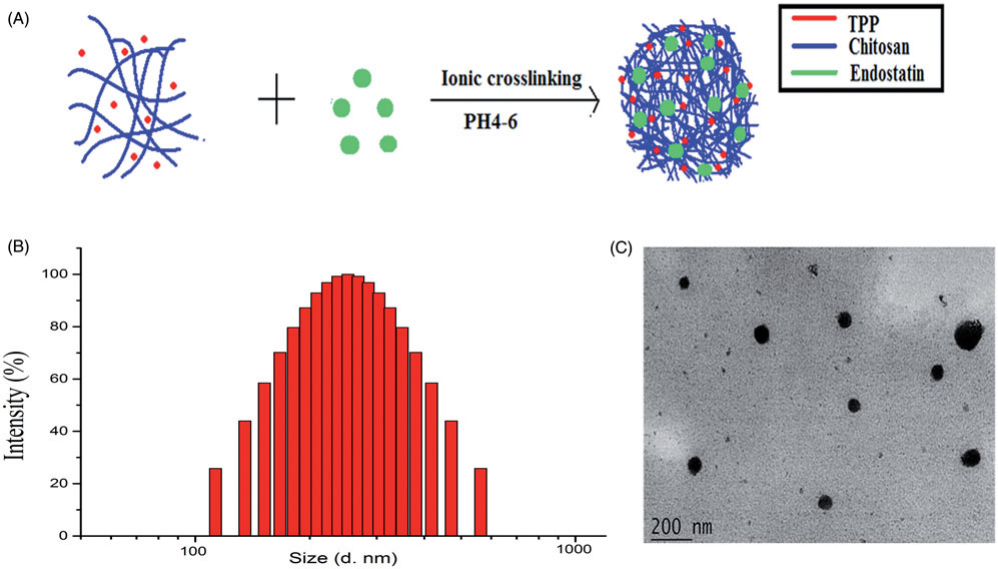
Characteristics of ES-loaded NPs. (A) The fabrication process of ES-NPs. ES-loaded chitosan nanoparticles were prepared using ionic cross-linking method with dropwise addition of TPP to a chitosan solution. (B) Size distribution of the ES-NPs. The results showed that the particles were 223.45 ± 5.1 nm in diameter. (C) TEM images of ES-NPs.

ES-NPs were prepared by ionic cross-linking with dropwise addition of TPP to a chitosan solution (Figure 1(A)). DL of these ES-NPs was 9.76 ± 0.24% and the EE was 71.03 ± 3.56%. The size and dispersity of the produced ES-NPs were confirmed by TEM morphology (Figure 1(B)), which revealed well-dispersed NPs into the aqueous solution. The particles were 223.45 ± 5.1 nm in diameter (Figure 1(C)). The protein dispersibility index (PDI) was 0.273 ± 0.006 and the zeta potential was 34.57 ± 0.12 mV.

### Release and stability of ES-loaded NPs *in vitro*

**Figure 2.**
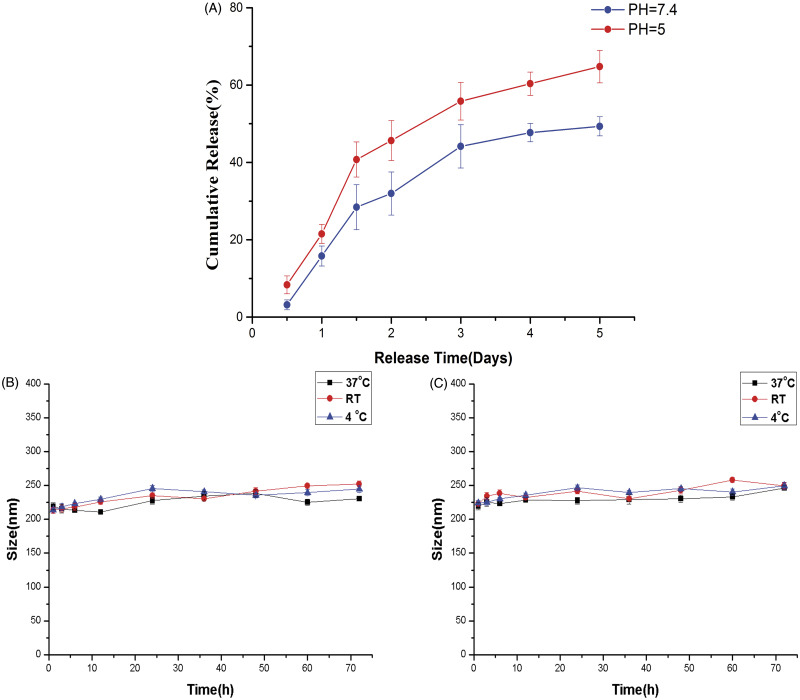
*In vitro* drug release of ES-NPs in two PBS solutions of different pH (A). The formulation stability of ES-NPs with (B) or without (C) mouse serum at 4 °C, 37 °C, or room temperature (RT).

The release of ES from the ES-NPs in PBS is shown in Figure 2(A). ES-NPs exhibited a rapid release behavior at pH 7.4 and 5, with 44.15 ± 3.12% and 54.75 ± 2.62% of the drug being released within 3 days, respectively. However, only 49.34 ± 2.48% ES was slowly released at pH 7.4 through 5 days. The results confirmed the biphasic nature of the ES release profile, with an initial abrupt release and a subsequent sustained release.

As shown in Figure 2(B) and (C), there was no obvious change in the mean diameter in each group, indicating that the NPs were stable in mouse serum and daily storage.

### *In vivo* tumor growth inhibition by ES-NPs

**Figure 3.**
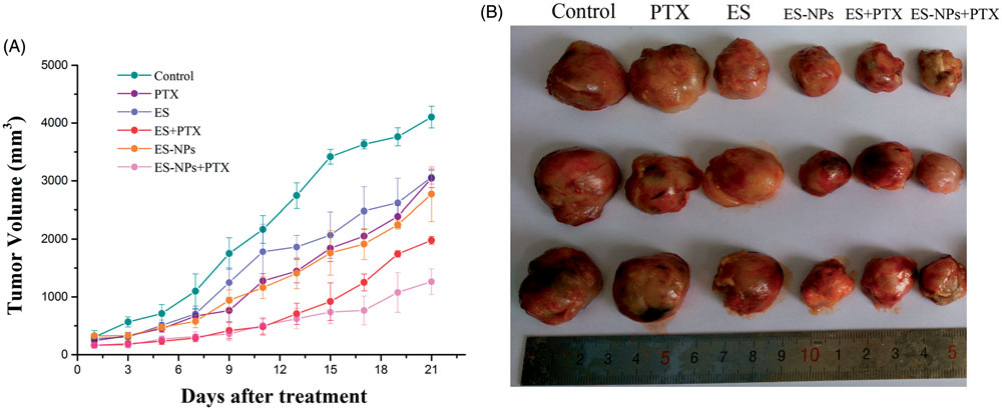
Tumor growth in subcutaneous Lewis lung cancer model. (A) Suppression of subcutaneous tumor growth in each group. (B) The final tumor volume on day 21.

To evaluate the effect of ES-NPs combined with PTX on LLC mouse xenografts, tumor volume was measured and plotted after each treatment. The curve of the experimental group was more gentle compared to the control group. After 21 days, the tumor volume of the ES-NPs + PTX group (1263.38 ± 220.68 mm^3^) was significantly smaller than the ES + PTX group (2772.21 ± 468 mm^3^) (*p* < .01), the PTX group (3042.50 ± 159.51 mm^3^, *p* < .01), the ES-NPs group (1980.25 ± 67.75 mm^3^, *p* < .01), the ES group (3061.53 ± 117.52 mm^3^, *p* < .01), and the control group (4102.38 ± 188.19 mm^3^, *p* < .01) (Figure 3(A)). The inhibition rate on day 21 was 70.27% in the ES-NPs + PTX group, 50.87% in the ES-NPs group, 33.83% in the ES + PTX group, and 24.73% in the ES group. These results demonstrated that ES-NPs + PTX was the most effective treatment in reducing the tumor volume (Figure 3(B)). There were no significant differences in the body weight of mice between the six groups, before and after treatment (data not shown).

### ELISA analysis

**Figure 4.**
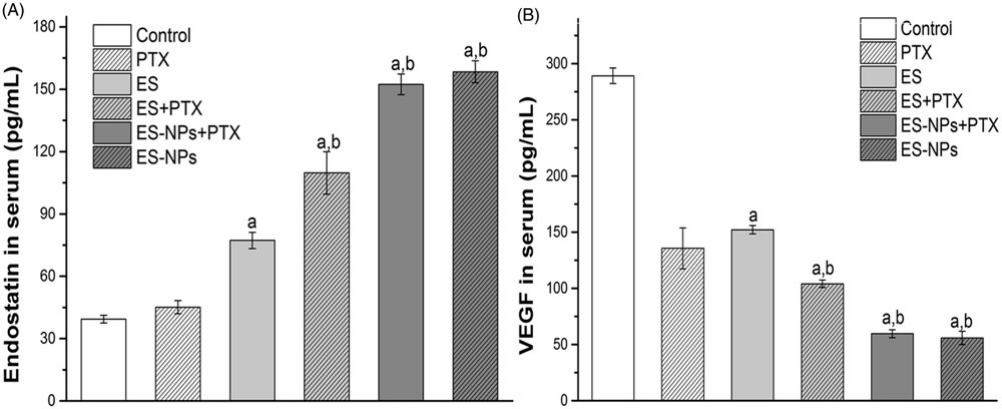
Serum ES (A) and VEGF (B) levels of each group. Mice in each group were sacrificed on day 21 and blood samples were collected to detect serum ES and VEGF levels by ELISA. ^a^*p* < .05 versus the control; ^b^*p* < .05 versus the ES group.

The serum levels of ES and VEGF are shown in Figure 4. The serum level of ES (Figure 4(A)) in the ES-NPs + PTX group was significantly higher than that detected in the control (*p* < .001), ES (*p* < .001), ES + PTX (*p* < .001), and PTX groups (*p* < .001), but was not significantly difference from the ES-NPs group (*p* = .141). Comparison of VEGF serum levels again revealed no significant difference between the ES-NPs + PTX and ES-NPs groups (*p* = .552). In addition, the level of VEGF in the ES-NPs + PTX group (Figure 4(B)) was significantly lower than the control (*p* < .001), ES (*p* < .001), ES + PTX (*p* < .001), and PTX groups (*p* < .001).

### Immunohistochemistry

**Figure 5.**
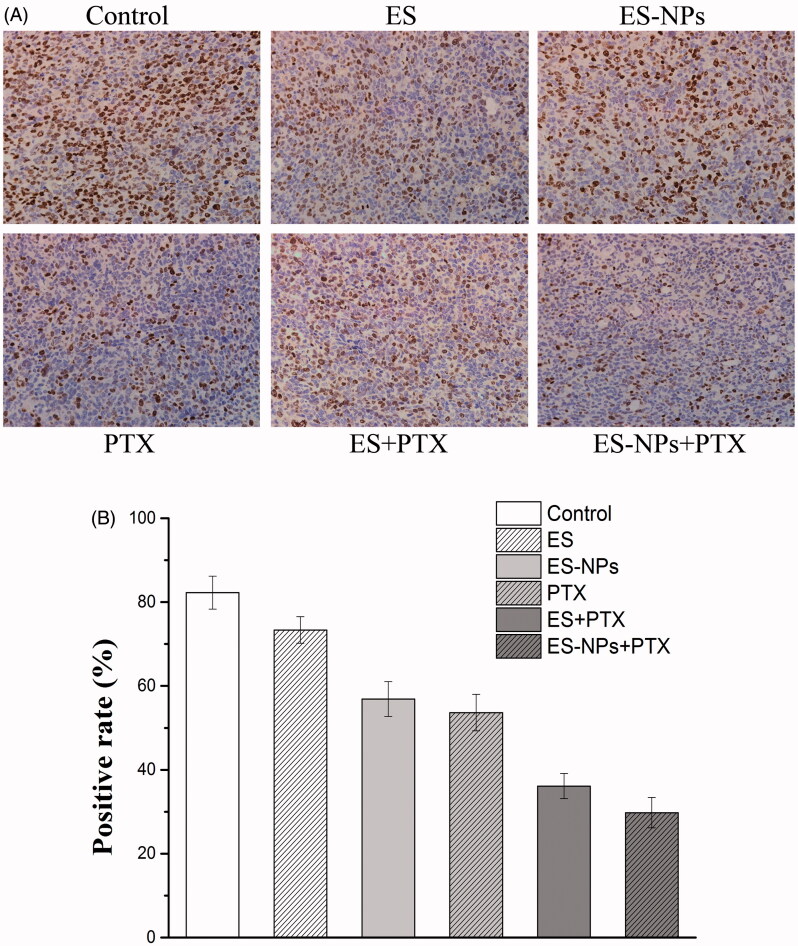
Ki-67 immunohistochemical staining in tumors. (A) Ki-67 immunohistochemical images of tumor tissue from mice in various groups. (B) Ki-67 quantitative analysis in xenografts from mice in various groups. Original magnification, 400×.

A significantly smaller number of Ki-67 positive cells (Figure 5) was observed in the ES-NPs + PTX group (29.78 ± 3.64%) compared to the PTX group (53.63 ± 4.35%, *p* < .05), the ES + PTX group (36.12 ± 2.99%, *p* < .05), the ES group (73.35 ± 3.16%, *p* < .05), the ES-NPs group (56.871 ± 4.16%, *p* < .05), and the control group (82.23 ± 3.93%, *p* < .05).

**Figure 6.**
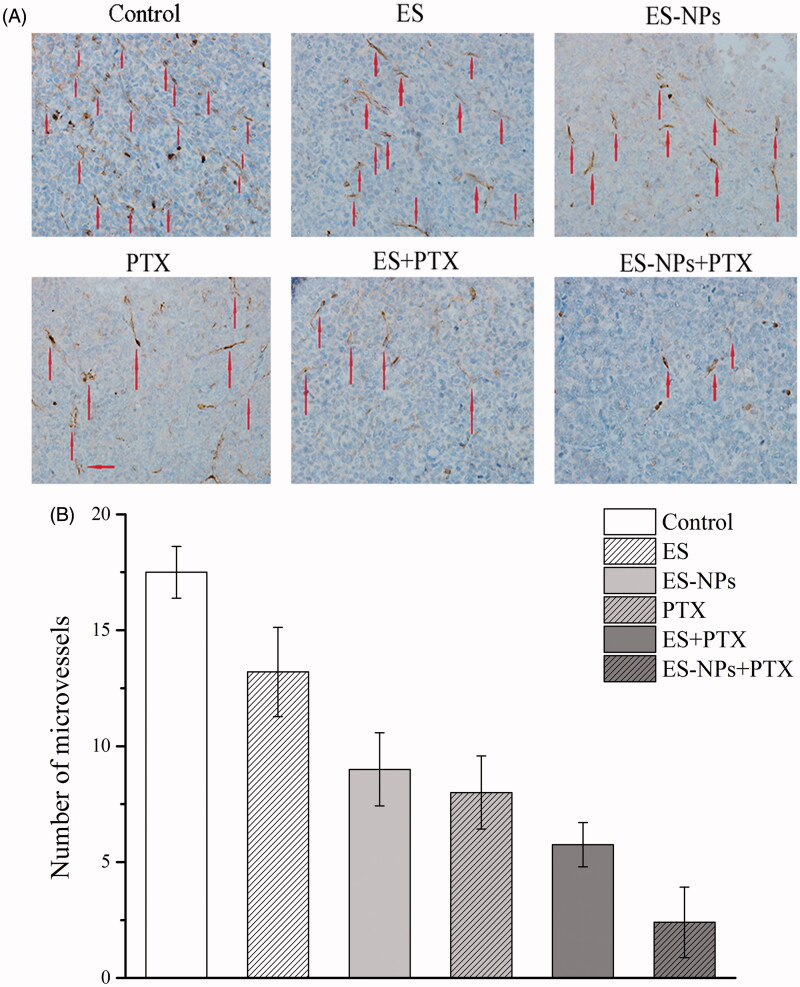
CD31 immunohistochemical staining of tumors. (A) CD31 immunohistochemical images of tumor tissue from mice in various groups. (B) CD31 quantitative analysis in xenografts from mice in various groups. Original magnification, 400×.

The MVD in each group was assessed by counting the number of CD31-positive cells. Fewer immune-reactive microvessels were observed in the tumor tissue sections of the ES-NPs + PTX treated mice (Figure 6), with MVD in the ES-NPs + PTX group (2.4 ± 1.51%) being significantly lower than the ES + PTX group (5.7 ± 0.96%, *p* < .05), the ES group (13.2 ± 1.92%, *p* < .05), the ES-NPs group (9 ± 1.58%, *p* < .05), the PTX group (8.0 ± 0.74%, *p* < .05), or the control group (17.5 ± 1.11%, *p* < .05).

## Discussion

In the present study, the method used to obtain ES-NPs allowed the preparation of NPs with a specific stability, high EE and drug loading, consistent with previous studies (Singh & Lillard, 2009; Amidi et al., 2010; Rampino et al., 2013). In particular, treatment with the ES-NPs overcame multiple limitations that included rapid elimination from the systemic circulation as a result of renal filtration and susceptibility to enzymatic degradation, the danger of developing an immune response and uptake by the reticuloendothelial system (Witting et al., 2015). The latter allows passage through the smallest capillary vessels and penetration of cells or access to the target site, as well as accumulation in certain solid tumors through the hyper-permeable vasculature that solid tumors exhibit. Furthermore, the particles can remain at the site because of the absence of tumor's lymphatic drainage (Cairns et al., 2006; Jain & Stylianopoulos, 2010; Chauhan et al., 2011).

*In vitro* release experiments with ES-NPs revealed a biphasic release profile, which agrees with prior results (Ding et al., 2017). On the one hand, an initial burst release rate in the first 36 h was observed in two PBS solutions of different pH, which might be due to the physical adsorption of peptides on the surface of the NPs. This phenomenon has also been observed with chitosan NPs loaded with PTX (Jiang et al., 2017), methotrexate, and mitomycin C (Jia et al., 2014). On the other hand, release of ES absorbed in chitosan occurred in a sustained manner during later time points, with the cumulative dissolution of ES reaching 49.34 ± 2.48% by 120 h. The relatively slow release of ES can be explained by strong electrostatic contacts and the slow hydrolysis of peptide bonds (Vandermeulen & Klok, 2004). In addition, abnormal microcirculation in tumors leads to a hostile microenvironment characterized by hypoxia and acidosis (Fukumura & Jain, 2007; Gillies et al., 2008), and the pH 5 PBS solution simulated this phenomenon. ES-NPs exhibited a slightly higher ES release at pH 5 than at pH 7.4, which may suggest that the protonation of the imidazole groups in the acidic environment leads to the hydrophilization of the hydrophobic core and partial release of the loaded chitosan nanoparticles.

The use of antiangiogenic agents has become an important treatment option for NSCLC patients. ES has proven its efficacy in treating advanced NSCLC patients when combined with chemotherapeutic agents. ES increase the clinical efficacy of chemotherapeutic agents, significantly prolongs disease progression time and improves the quality of life of the patients (Wang et al., 2005; Rong et al., 2012; Zhu et al., 2015). The antitumor effect of ES is improved when incorporated into chitosan NPs (Ding et al., 2017). ES-NPs were more effective than ES in our LLC model. In our *in vivo* experiments, we used a lung cancer xenograft model to determine whether ES-NPs combined with PTX improved tumor growth inhibition compared to ES combined with PTX. As anticipated, at equivalent ES doses, ES-NPs combined with PTX significantly improved tumor growth inhibition compared to direct ES injection in mice, with a tumor inhibition rate of 70.27%. We speculate that ES-NPs extend the retention time of ES *in vivo* and enhance the inhibition of tumor blood vessels. Although the ES + PTX group also displayed a good curative effect over the first 14 days, due to the shorter retention time of ES, tumor angiogenesis tumor angiogenesis was reactivated, resulting in rapid proliferation of the tumor cells. The results were confirmed by serum and immunohistochemical analyzes.

The MVD and Ki-67 in the ES-NPs + PTX group were significantly lower than in the ES + PTX group (*p* < .05), which could have been caused by better suppression of cell proliferation and angiogenesis. Furthermore, the serum ES levels in the ES-NPs and ES-NPs + PTX groups were significantly higher than those detected in the serum of the other groups (*p* < .05, Figure 4(A)), whereas the serum VEGF levels in the former two groups were markedly lower than the other groups (*p* < .05, Figure 4(B)). This indicates that ES-NPs significantly prolong the retention time of ES *in vivo*. After 7 days of treatment, ES, which has been confirmed to block the VEGF-induced tyrosine phosphorylation of KDR/Flk-1 and down-regulate the expression of VEGF (Kim et al., 2002; Abdollahi et al., 2003; Ling et al., 2007), could reduce the protein expression of VEGF and still remain at high levels in the serum, which could explain the low MVD observed in the ES-NPs + PTX and ES-NPs groups. However, the ES-NPs + PTX group displayed much stronger inhibition of tumor blood vessels than the ES-NPs group. Consequently, we infer that PTX kills tumor cells and simultaneously reduces the autocrine functions of VEGF, which would further suppress angiogenesis.

In this study, the ES-NPs + PTX group exhibited significant antitumor and antiangiogenic effects. This suggests that ES-NPs increase the stability and the concentration of ES in the blood. Moreover, ES-NPs enhanced the antitumor effect of PTX without increasing toxicity. Although the ES-NPs + PTX group showed the strongest inhibitory effect on LLC, based on the vascular normalization time window, the optimal administration plan must be determined in further studies.

## Conclusions

A chitosan NPs drug delivery system loaded with ES was prepared using ionic cross-linking and its’ *in vivo* anti-tumor activity was investigated in a LLC mouse model. The NPs were prepared using the solid dispersion method and resulted in a compound with high DL, high EE, and spherical morphology. *In vitro* drug release experiments showed that ES-NPs could release ES in a controlled manner. *In vivo*, ES-NPs combined with PTX significantly inhibited tumor growth. Analysis of tissue biomarkers showed that the ES-NPs combined with PTX suppressed tumor cell proliferation and inhibited angiogenesis in the tumor tissue. Thus, the results indicate that ES-NPs can overcome the shortcomings of free ES, such as short retention time in circulation, resulting in an enhanced antitumor activity of ES. Moreover, the antitumor effect can be strengthened further by combining the ES-NPs with a cytotoxic agent, like PTX.

## References

[CIT0001] Abdollahi A, Lipson KE, Sckell A, et al. (2003). Combined therapy with direct and indirect angiogenesis inhibition results in enhanced antiangiogenic and antitumor effects. Cancer Res 63:8890–8. 14695206

[CIT0002] Al-Qadi S, Grenha A, Carrión-Recio D, et al. (2012). Microencapsulated chitosan nanoparticles for pulmonary protein delivery: in vivo evaluation of insulin-loaded formulations. J Control Release 157:383–90. 21864592 10.1016/j.jconrel.2011.08.008

[CIT0003] Amidi M, Mastrobattista E, Jiskoot W, et al. (2010). Chitosan-based delivery systems for protein therapeutics and antigens. Adv Drug Deliv Rev 62:59–82. 19925837 10.1016/j.addr.2009.11.009

[CIT0004] Azzoli CG, Temin S, Giaccone G. (2012). 2011 Focused update of 2009 American Society of Clinical Oncology clinical practice guideline update on chemotherapy for stage IV non-small-cell lung cancer. J Oncol Pract 8:63–6. 22548014 10.1200/JOP.2011.000374 PMC3266319

[CIT0005] Cairns R, Papandreou I, Denko N. (2006). Overcoming physiologic barriers to cancer treatment by molecularly targeting the tumor microenvironment. Mol Cancer Res 4:61–70. 16513837 10.1158/1541-7786.MCR-06-0002

[CIT0006] Calvo P, Remuñan-López C, Vila-Jato JL, Alonso MJ. (1997). Chitosan and chitosan/ethylene oxide-propylene oxide block copolymer nanoparticles as novel carriers for proteins and vaccines. Pharm Res 14:1431–6. 9358557 10.1023/a:1012128907225

[CIT0007] Chauhan VP, Stylianopoulos T, Boucher Y, et al. (2011). Delivery of molecular and nanoscale medicine to tumors: transport barriers and strategies. Annu Rev Chem Biomol Eng 2:281–98. 22432620 10.1146/annurev-chembioeng-061010-114300

[CIT0008] Chen W, Hu S. (2011). Suitable carriers for encapsulation and distribution of endostar: comparison of endostar-loaded particulate carriers. Int J Nanomedicine 6:1535–41. 21845043 10.2147/IJN.S21881 PMC3152471

[CIT0009] Ding RL, Xie F, Hu Y, et al. (2017). Preparation of endostatin-loaded chitosan nanoparticles and evaluation of the antitumor effect of such nanoparticles on the Lewis lung cancer model. Drug Deliv 24:300–8. 28165807 10.1080/10717544.2016.1247927 PMC8241108

[CIT0010] Ferrara N, Kerbel RS. (2005). Angiogenesis as a therapeutic target. Nature 438:967–74. 16355214 10.1038/nature04483

[CIT0011] Fukumura D, Jain RK. (2007). Tumor microvasculature and microenvironment: targets for anti-angiogenesis and normalization. Microvasc Res 74:72–84. 17560615 10.1016/j.mvr.2007.05.003 PMC2100036

[CIT0012] Gao J, Knutsen A, Arbman G, et al. (2009). Clinical and biological significance of angiogenesis and lymphangiogenesis in colorectal cancer. Dig Liver Dis 41:116–22. 19038587 10.1016/j.dld.2008.07.315

[CIT0013] Gillies RJ, Robey I, Gatenby RA. (2008). Causes and consequences of increased glucose metabolism of cancers. J Nucl Med 49:24S–42s. 18523064 10.2967/jnumed.107.047258

[CIT0014] Han B, Xiu Q, Wang H, et al. (2011). A multicenter, randomized, double-blind, placebo-controlled study to evaluate the efficacy of paclitaxel-carboplatin alone or with endostar for advanced non-small cell lung cancer. J Thorac Oncol 6:1104–9. 21532504 10.1097/JTO.0b013e3182166b6b

[CIT0015] Jain RK, Stylianopoulos T. (2010). Delivering nanomedicine to solid tumors. Nat Rev Clin Oncol 7:653–64. 20838415 10.1038/nrclinonc.2010.139 PMC3065247

[CIT0016] Jia M, Li Y, Yang X, et al. (2014). Development of both methotrexate and mitomycin C loaded PEGylated chitosan nanoparticles for targeted drug codelivery and synergistic anticancer effect. ACS Appl Mater Interfaces 6:11413–23. 24977925 10.1021/am501932s

[CIT0017] Jiang J, Liu Y, Wu C, et al. (2017). Development of drug-loaded chitosan hollow nanoparticles for delivery of paclitaxel to human lung cancer A549 cells. Drug Dev Ind Pharm 43:1304–13. 28402175 10.1080/03639045.2017.1318895

[CIT0018] Kim YM, Hwang S, Kim Y-M, et al. (2002). Endostatin blocks vascular endothelial growth factor-mediated signaling via direct interaction with KDR/Flk-1. J Biol Chem 277:27872–9. 12029087 10.1074/jbc.M202771200

[CIT0019] Klose R, Krzywinska E, Castells M, et al. (2016). Targeting VEGF-A in myeloid cells enhances natural killer cell responses to chemotherapy and ameliorates cachexia. Nat Commun 7:12528. 27538380 10.1038/ncomms12528 PMC4992172

[CIT0020] Lee KY, Ha WS, Park WH. (1995). Blood compatibility and biodegradability of partially N-acylated chitosan derivatives. Biomaterials 16:1211–16. 8589189 10.1016/0142-9612(95)98126-y

[CIT0021] Li T, Kung H-J, Mack PC, et al. (2013). Genotyping and genomic profiling of non-small-cell lung cancer: implications for current and future therapies. J Clin Oncol 31:1039–49. 23401433 10.1200/JCO.2012.45.3753 PMC3589700

[CIT0022] Ling Y, Yang Y, Lu N, et al. (2007). Endostar, a novel recombinant human endostatin, exerts antiangiogenic effect via blocking VEGF-induced tyrosine phosphorylation of KDR/Flk-1 of endothelial cells. Biochem Biophys Res Commun 361:79–84. 17644065 10.1016/j.bbrc.2007.06.155

[CIT0023] Mukherjee B, Santra K, Pattnaik G, Ghosh S. (2008). Preparation, characterization and in-vitro evaluation of sustained release protein-loaded nanoparticles based on biodegradable polymers. Int J Nanomed 3:487–96. 10.2147/ijn.s3938 PMC2636584 19337417

[CIT0024] Nasti A, Zaki NM, de Leonardis P, et al. (2009). Chitosan/TPP and chitosan/TPP-hyaluronic acid nanoparticles: systematic optimisation of the preparative process and preliminary biological evaluation. Pharm Res 26:1918–30. 19507009 10.1007/s11095-009-9908-0

[CIT0025] O'Reilly MS, Boehm T, Shing Y, et al. (1997). Endostatin: an endogenous inhibitor of angiogenesis and tumor growth. Cell 88:277–85. 9008168 10.1016/s0092-8674(00)81848-6

[CIT0026] Rampino A, Borgogna M, Blasi P, et al. (2013). Chitosan nanoparticles: preparation, size evolution and stability. Int J Pharm 455:219–28. 23886649 10.1016/j.ijpharm.2013.07.034

[CIT0027] Rong B, Shuanying Y, Wei L, et al. (2012). Systematic review and meta-analysis of Endostar (rh-endostatin) combined with chemotherapy versus chemotherapy alone for treating advanced non-small cell lung cancer. World J Surg Oncol 10:170. 22917490 10.1186/1477-7819-10-170 PMC3517896

[CIT0028] Shi S, Chen L, Huang G. (2013). Antiangiogenic therapy improves the antitumor effect of adoptive cell immunotherapy by normalizing tumor vasculature. Med Oncol 30:698. 23982676 10.1007/s12032-013-0698-1

[CIT0029] Siegel RL, Miller KD, Jemal A. (2017). Cancer statistics, 2017. CA Cancer J Clin 67:7–30. 28055103 10.3322/caac.21387

[CIT0030] Singh R, Lillard JW Jr. (2009). Nanoparticle-based targeted drug delivery. Exp Mol Pathol 86:215–23. 19186176 10.1016/j.yexmp.2008.12.004 PMC3249419

[CIT0031] Sun XJ, Deng Q-H, Yu X-M, et al. (2016). A phase II study of endostatin in combination with paclitaxel, carboplatin, and radiotherapy in patients with unresectable locally advanced non-small cell lung cancer. BMC Cancer 16:266. 27067521 10.1186/s12885-016-2234-0 PMC4828797

[CIT0032] Swierczewska M, Han HS, Kim K, et al. (2016). Polysaccharide-based nanoparticles for theranostic nanomedicine. Adv Drug Deliv Rev 99:70–84. 26639578 10.1016/j.addr.2015.11.015 PMC4798864

[CIT0033] Takahashi O, Komaki R, Smith PD, et al. (2012). Combined MEK and VEGFR inhibition in orthotopic human lung cancer models results in enhanced inhibition of tumor angiogenesis, growth, and metastasis. Clin Cancer Res 18:1641–54. 22275507 10.1158/1078-0432.CCR-11-2324 PMC3306446

[CIT0034] Tentler JJ, Bradshaw-Pierce EL, Serkova NJ, et al. (2010). Assessment of the in vivo antitumor effects of ENMD-2076, a novel multitargeted kinase inhibitor, against primary and cell line-derived human colorectal cancer xenograft models. Clin Cancer Res 16:2989–98. 20406842 10.1158/1078-0432.CCR-10-0325 PMC3928713

[CIT0035] Torchilin VP, Lukyanov AN. (2003). Peptide and protein drug delivery to and into tumors: challenges and solutions. Drug Discov Today 8:259–66. 12623240 10.1016/s1359-6446(03)02623-0

[CIT0036] Torre LA, Bray F, Siegel RL, et al. (2015). Global cancer statistics, 2012. CA Cancer J Clin 65:87–108. 25651787 10.3322/caac.21262

[CIT0037] Vandermeulen GW, Klok HA. (2004). Peptide/protein hybrid materials: enhanced control of structure and improved performance through conjugation of biological and synthetic polymers. Macromol Biosci 4:383–98. 15468229 10.1002/mabi.200300079

[CIT0038] Wang J, Sun Y, Liu Y, et al. (2005). Results of randomized, multicenter, double-blind phase III trial of rh-endostatin (YH-16) in treatment of advanced non-small cell lung cancer patients. Zhongguo Fei Ai Za Zhi 8:283–90. 21108883 10.3779/j.issn.1009-3419.2005.04.07

[CIT0039] Witting M, Obst K, Friess W, et al. (2015). Recent advances in topical delivery of proteins and peptides mediated by soft matter nanocarriers. Biotechnol Adv 33:1355–69. 25687276 10.1016/j.biotechadv.2015.01.010

[CIT0040] Xu Y, Strickland EC, Fitzgerald MC. (2014). Thermodynamic analysis of protein folding and stability using a tryptophan modification protocol. Anal Chem 86:7041–8. 24896224 10.1021/ac501278j

[CIT0041] Yu ZW, Ju Y-H, Yang C-L, et al. (2015). Antitumor effect of recombinant human endostatin combined with cisplatin on rats with transplanted Lewis lung cancer. Asian Pac J Trop Med 8:664–7. 26321522 10.1016/j.apjtm.2015.07.010

[CIT0042] Zhu Q, Zang Q, Jiang Z-M, et al. (2015). Clinical application of recombinant human endostatin in postoperative early complementary therapy on patients with non-small cell lung cancer in Chinese Mainland. Asian Pac J Cancer Prev 16:4013–18. 25987078 10.7314/apjcp.2015.16.9.4013

